# Effects of Very Low Calorie Ketogenic Diet on the Orexinergic System, Visceral Adipose Tissue, and ROS Production

**DOI:** 10.3390/antiox8120643

**Published:** 2019-12-13

**Authors:** Anna Valenzano, Rita Polito, Valentina Trimigno, Antonella Di Palma, Fiorenzo Moscatelli, Gaetano Corso, Francesco Sessa, Monica Salerno, Angelo Montana, Nunzio Di Nunno, Marinella Astuto, Aurora Daniele, Marco Carotenuto, Giovanni Messina, Giuseppe Cibelli, Vincenzo Monda

**Affiliations:** 1 Department of Clinical and Experimental Medicine, University of Foggia, 71122 Foggia, Italy; anna.valenzano@unifg.it (A.V.); rita.polito@unicampania.it (R.P.); valentina_trimigno.549305@unifg.it (V.T.); antonella_dipalma.549441@unifg.it (A.D.P.); fiorenzo400@gmail.com (F.M.); gaetano.corso@unifg.it (G.C.); francesco.sessa@unifg.it (F.S.); giovanni.messina@unifg.it (G.M.); 2 Department of Medical, Surgical and Advanced Technologies “G.F. Ingrassia”, University of Catania, 95123 Catania, Italy; salerno.monica@gmail.com (M.S.); angelomontana49@gmail.com (A.M.); 3 Department of History, Society and Studies on Humanity, University of Salento, 73100 Lecce, Italy; nunzio.dinunno@unisalento.it; 4 Department of Biomedical and Biotechnological Sciences, University of Catania, 95123 Catania, Italy; marinella.astuto@policlinico.unict.it; 5 CEINGE Biotecnologie Avanzate S.C. a r.l., 80131 Napoli, Italy; aurora.daniele@unicampania.it; 6 Clinic of Child and Adolescent Neuropsychiatry, Department of Mental Health, Physical and Preventive Medicine, Università degli Studi della Campania “Luigi Vanvitelli”, 80100 Naples, Italy; marco.carotenuto@unicampania.it; 7 Department of Experimental Medicine, Section of Human Physiology and Unit of Dietetics and Sports Medicine, Università degli Studi della Campania “Luigi Vanvitelli”, 80100 Naples, Italy; vincenzo.monda@unicampania.it

**Keywords:** VLCK diet, orexin-A, visceral adipose tissue

## Abstract

Background: Caloric restriction is a valid strategy to reduce the visceral adipose tissue (VAT) content in obese persons. Hypocretin-1 (orexin-A) is a neuropeptide synthesized in the lateral hypothalamus that strongly modulates food intake, thus influencing adipose tissue accumulation. Therapeutic diets in obesity treatment may combine the advantages of caloric restriction and dietary ketosis. The current study aimed to evaluate the effect of a very low calorie ketogenic diet (VLCKD) in a population of obese patients. Methods: Adiposity parameters and orexin-A serum profiling were quantified over an 8 week period. The effect of the VLCKD on reactive oxygen species (ROS) production and cell viability was evaluated, in vitro, by culturing Hep-G2 cells in the presence of VLCKD sera. Results: Dietary intervention induced significant effects on body weight, adiposity, and blood chemistry parameters. Moreover, a selective reduction in VAT was measured by dual-energy X-ray absorptiometry. Orexin-A levels significantly increased after dietary treatment. Hep-G2 cell viability was not affected after 24, 48, and 72 h incubation with patients’ sera, before and after the VLCKD. In the same model system, ROS production was not significantly influenced by dietary treatment. Conclusion: The VLCKD exerts a positive effect on VAT decrease, ameliorating adiposity and blood chemistry parameters. Furthermore, short-term mild dietary ketosis does not appear to have a cytotoxic effect, nor does it represent a factor capable of increasing oxidative stress. Finally, to the best of our knowledge, this is the first study that shows an effect of the VLCKD upon the orexinergic system, supporting the usefulness of such a therapeutic intervention in promoting obesity reduction in the individual burden of this disease.

## 1. Introduction

Obesity is a long-term multifactorial chronic disease characterized by an energy imbalance due to an excess of caloric intake compared with energy expenditure and the deregulation of other metabolic parameters, such as altered lipid profile, increased insulin resistance, and chronic proinflammatory state [1]. Obesity is associated with a major risk of developing chronic diseases, such as type 2 diabetes, hypertension, hyperlipidemia, cardiovascular diseases, cerebrovascular accidents, and musculoskeletal disorders, and with an increased risk of cancer, particularly for the esophagus, colorectum, breast, endometrium, and kidney [2]. Obesity types may be distinguished between central (adipose tissue around the abdomen rather than appendicular accumulation) and visceral. The resulting central adipose tissue is composed of both subcutaneous adipose tissue (SAT) and visceral adipose tissue (VAT) compartments. The severity of obesity-related diseases is closely linked to visceral adiposity, considering that VAT may cause a subclinical systemic inflammation condition [3]. Visceral adiposity is also associated with metabolic syndrome and markers of insulin resistance [4,5,6]. As a metabolically active organ, VAT communicates with other central and peripheral organs by synthesizing and secreting a number of molecules, generally referred to as adipokines, directly linked to metabolic homeostasis, which highlights the central role of adipose tissue in energy homeostasis regulation [7,8]. Obesity is also characterized by an imbalance in the neurochemical pathways, particularly orexin-A/hypocretin-1 (ORX1) synthesized in the lateral hypothalamus and involved in the regulation of appetite, metabolic rate, energy expenditure, social development, and pain and sleep regulation [9,10,11,12]. Orexin-A is also involved in the regulation of inflammatory response, acting as an anti-inflammatory agent [13]. In obese subjects’ sera, orexin-A levels are strongly reduced and inversely correlated with proinflammatory mediators such as C-reactive protein (CRP) and body mass index (BMI) [14]. Previous studies showed that in human adipocytes isolated from subcutaneous, compared with intra-abdominal, adipose tissue, orexin-A is able to reduce adipogenesis in intra-abdominal but not subcutaneous adipocytes [15]. These results have been recently reinforced by the evidence of a small but significant reduction of visceral fat mass (FM) in orexin-A-treated mice not paralleled by differences in the subcutaneous fat, suggesting a lower sensitivity to orexin-A [16]. 

Caloric restriction reduces or slows the onset of diseases related to obesity, inducing a considerable weight loss and having beneficial anti-inflammatory effects, reducing the production of free radicals, and favoring greater resistance to stress and a prolonged lifespan [17]. Correct nutrition and regular physical activity are able to activate numerous metabolic pathways such as SIRT1, which downregulates the production of mediators of inflammation and reduces reactive oxygen species (ROS) production [18]. In the light of this, a caloric restriction diet can be an efficient therapeutic approach to promote weight loss in obese patients, although conflicting results have been reported about the ideal composition for nutritional therapy against obesity [19]. 

Specifically, a very low carbohydrate diet (usually ≤50 g/day) is a dietetic regimen mimicking fasting by reducing caloric intake and restricting carbohydrates and fats that is proposed to achieve rapid weight loss [20]. However, mild dietary ketosis is a physiological mechanism; the ketogenic diet, inducing a large production of ketone bodies, may be a therapeutic strategy in obesity-related and cardiovascular disease. In fact, apart from serving as an energy fuel, ketone bodies play pivotal roles as signaling mediators, drivers of protein post-translational modification, and modulators of inflammation and oxidative stress. A very low carbohydrate diet is generally considered an equivalent of the ketogenic diet. Many reports from the literature show that a very low carbohydrate ketogenic diet (VLCKD) can ameliorate the lipid profile and reduce some cardiovascular risk factors. Furthermore, this dietary intervention may also play a role in cancer therapy by enhancing its therapeutic responses. The VLCKD has been shown to be effective in the short to medium term (3–6 months) as a tool to counter obesity [18,19,20]. The VLCKD is more effective in inducing weight loss compared with a standard low-calorie diet (LCD), presenting higher patient compliance as well as only mild and transitory side effects, but the effects of an LCD are more lasting over time. Indeed, the current problem with fighting obesity is not only losing weight but also maintaining weight loss over time. 

The aim of the present study was to quantify the effects of a commercial weight-loss dietary ketosis program on visceral fat mass, evaluated by dual-energy X-ray absorptiometry (DXA), and the impact of 8 weeks of treatment on orexin-A production and reactive oxygen metabolite (d-ROM) levels in vivo, in a population of obese patients treated with the VLCKD, and in vitro, in Hep-G2 cells, to evaluate cell viability and oxidative stress in response to treatment with VLCKD sera before and after the weight-loss dietary ketosis program. 

## 2. Materials and Methods 

### 2.1. Participants

Twenty obese subjects (10 females and 10 males), aged between 20–60 years (mean 48 ± 10 years), volunteered to participate in the study. All enrolled females were not in menopause. The study took place at the Laboratory of Physiology, Department of Clinical and Experimental Medicine, University of Foggia. Written informed consent for participation in the study was obtained before recruiting. This study was performed in accordance with the Declaration of Helsinki and approved by the local ethics committee (no. 440/DS). Participants were excluded if they had a prior medical history of renal insufficiency, hyperuricemia, severe hepatic insufficiency, type 1 or 2 diabetes mellitus treated with insulin, atrioventricular block, heart failure, cardiovascular and cerebrovascular diseases, unbalanced hypokalemia, hypo- or hyperthyroidism, chronic treatment with corticosteroid drugs, severe mental disorders, neoplasms, pregnancy, or they were lactating. 

### 2.2. Study Protocol

The study followed an open, controlled, 8 week nutritional intervention design, with study examinations at baseline and week 8. A control group was not included. All participants were highly motivated and none of them had any previous experience with or preconceptions about low carbohydrate or ketogenic diets. Prior to the start of the dietary intervention, participants underwent a general medical examination. Baseline variables included age, gender, height, weight, blood pressure, and laboratory tests. During the study, dietary adherence was measured daily, between 2:00 and 4:00 p.m., by capillary blood ketone assessment (GD40 Delta test strips, TaiDoc Technology Co., Taiwan). Nutritional ketosis is defined as a blood ketone (β-hydroxybutyrate) level > 0.5 mmol/L. Body weight was recorded at the same time each day to the nearest 0.1 kg (SECA 711, Hamburg, Germany) with the subject wearing light clothing, and height was recorded to the nearest 0.1 cm (SECA 213, Hamburg, Germany). Blood tests were taken at baseline and week 8 after a 12 h fast. Fasting blood samples were collected at 8:00 a.m. from an antecubital vein using a 21G Vacutainer blood collection set (BD Diagnostics, Franklin Lakes, NJ, USA). Blood samples were centrifuged and the resultant serum stored at −80 °C until use. 

### 2.3. Orexin-A Assay

Plasma orexin-A concentrations were determined by an enzyme-linked immunoassay (ELISA), using a commercial kit according to the manufacturer’s instructions (Phoenix Pharmaceuticals, USA). No significant cross-reactivity or interference between orexin-A and analogues were described. Briefly, Sep-Pak C18 columns (Waters, Milford, MA, USA) were used to extract orexin-A from obese subjects’ sera before and after dietary ketosis. Columns were activated by adding 10 mL of methanol and 20 mL of H_2_O, charged with 1–2 mL samples and washed with 20 mL of H_2_O. The columns were then eluted with 80% acetonitrile and the resulting eluate was reduced to 400 µL under nitrogen flow. The dry residue, obtained by Speedvac evaporation (Savant Instruments, Holbrook, NY, USA), was dissolved in water and used for a further ELISA assay. No cross-reactivity between orexin-A (16–33), orexin-B, and agouti-related protein (83–132) amide was detected. The minimal detectable concentration was 0.37 ng/mL. Intra- and interassay errors were <5% and <14%, respectively.

Body Composition and Selective Visceral Fat Mass Analysis

Body composition was estimated by DXA (Lunar Prodigy DXA, GE Healthcare, Madison, WI, USA) via whole-body scan. Visceral Adipose Tissue (VAT) was quantified by subtracting subcutaneous fat from total abdominal fat, reported in grams, using the CoreScan software package (GE Healthcare, Madison, WI, USA) [21].

### 2.4. Dietary Intervention

Participants followed the VLCKD according to a commercial weight-loss program (Lignaform, Therascience), consisting of <50 g/day carbohydrate from vegetables, 43% fat, 43% protein, 14% carbohydrates, and 700–900 kcal. The amount of protein ranged between 1.0 and 1.2 g/kg of ideal body weight. However, the dietary intervention profile consisted of three different stages; for the purposes of this study, only active ketogenic phases of the first stage were considered. This stage had an 8 week duration to allow participants to achieve approximately 80% of their weight-loss target. Over this period, vitamins, minerals, and omega-3 fatty acids were provided in accordance with international recommendations [22].

### 2.5. Cell Culture and Cell Proliferation Assay

The Hep-G2 cell line, derived from hepatocellular carcinoma, was purchased from the American Type Culture Collection (ATTC, LGC Standards srl, Sesto San Giovanni (MI), Italy). Cells were seeded at 4 × 10^3^ cells/well in 96-well plates and cultured at 37 °C in 5% CO_2_ in DMEM (Sigma-Aldrich, Milano, Italy), supplemented with 2 mM l-glutamine (Sigma-Aldrich, Italy), 100 U/mL penicillin/streptomycin, and 10% (*v/v*) fetal bovine serum (FBS) (Euroclone, Italy). Cultures were incubated at different time intervals (24, 48, and 72 h) in the presence of either 10% (*v/v*) pooled sera from VLCKD subjects, collected prior to and after dietary ketosis, or 10% (*v/v*) of FBS serum as control. After 24, 48, and 72 h of incubation, cell viability was analyzed by a Cell Proliferation Assay Kit (MTT- Sigma-Aldrich, Milano, Italy), following the manufacturer’s instructions. At the end of sera incubation, 10 μL of MTT labeling reagent was added and the cells were incubated for 4 h at 37 °C in a humidified atmosphere. At the end of 4 h, 100 μL of solubilization solution was added and incubated overnight at 37 °C in a humidified atmosphere. After overnight incubation, the spectrophotometric absorbance of the samples was measured using a microplate (ELISA) reader (Sigma-Aldrich, Milano, Italy). The wavelength to measure the absorbance of the formazan product was 550 nm. The experiments were performed in triplicate. 

### 2.6. ROS Production Assay

As reported in the previous section, Hep-G2 cells were seeded at 4 × 10^3^ cells/well in 96-well plates and cultured at 37 °C in 5% CO_2_ at different time intervals (24, 48, and 72 h) in the presence of either 5% (*v/v*) pooled sera from VLCKD subjects, collected prior to and after dietary ketosis, or 5% (*v/v*) of FBS serum as control. To evaluate ROS production in Hep-G2 cells, we added an ROS inhibitor, N-acetylcysteine (NAC), to negative control cells and incubated them for 60 min. After 24, 48, and 72 h of incubation, the direct impact of sera treatment on cell production of ROS was analyzed using the DCFDA Cellular ROS Detection Assay Kit (Abcam, Cambridge, UK), following the manufacturer’s instructions. DCFDA was added in the dark at a final concentration of 20 μm at 30 min before the end of sera incubation. DCFDA was added for 45 min at 37 °C. At the end of the incubation, the cells were collected and resuspended in 500 μL of PBS for fluorometric analysis. The excitation and emission wavelengths used were 485 and 535 nm, respectively. 

### 2.7. d-ROM Test

The d-ROM test via Fenton’s reaction was performed in the plasma of VLCKD participants before and after diet therapy. The peripheral blood sample was collected from the subject’s finger in a heparinized microcuvette and plasma was immediately isolated by centrifugation at 37 °C for 60 s. The plasma was dissolved in an acidic buffer (pH 4.8) in which its hydroperoxides react with the transition metal ions and were converted to alkoxy and peroxy radicals. Successively, N,N-diethyl-para-phenylenediamine was added, which was oxidized by these newly formed radicals, producing a magenta-colored derivative. The color variation was measured by a photometer at 505 nm and 37 °C. This color variation is directly correlated with the concentration of H_2_O_2_ in the plasma sample, which is proportional to the quantity of ROMs, according to the Beer-Lambert law. The d-ROM value was expressed in the arbitrary unit U. CARR (Units Carratelli), as established by the manufacturer (1 U. CARR corresponds to 0.08 mg of H_2_O_2_/dL). Normal values range between 250 and 300 U. CARR and values higher than 300 U. CARR suggest increased oxidative stress [23].

### 2.8. Statistical Analysis

ANOVA analysis was performed to verify the change from baseline to 8 weeks after VLCKD intervention, and post hoc pairwise comparisons were performed with Tukey’s honestly significant difference test. Distribution of continuous data was checked for normality (data were distributed normally). VAT, CRP, and orexin-A serum concentrations were correlated by Pearson’s test according to data distribution. Statistical analyses were performed using the StatView software package 5.0.1.0 (SAS Institute Inc., Cary, USA). All data are presented as mean ± standard error (SE). A *p*-value ≤ 0.05 was used for statistical significance.

## 3. Results 

### 3.1. Anthropometric and Biochemical Parameters of VLCKD Obese Patients

Our results show a significant change in the anthropometric and biochemical parameters of VLCKD obese subjects before and after diet therapy. Anthropometric parameters, such as weight and BMI, were statistically reduced in VLCKD obese subjects before and after diet (Table 1). As shown in Table 1, there was a statistically significant reduction of low-density lipoprotein (LDL), triglycerides, insulinemia, aspartate transaminase/glutamic oxaloacetic transaminase (AST/GOT), alanine transaminase/glutamic pyruvic transaminase (ALT/GPT), and gamma glutamyl transferase (GT), indicating a strong modulation and modification of glycemic and lipid profiles by VLCKD therapy in these subjects.

Furthermore, there was not only weight loss and BMI reduction, but as reported in Table 2, there was a significant reduction of FM and VAT (Table 2).

The modification of body composition and biochemical parameters also influenced a modification of cardiac parameters, such as diastolic and systolic arterial pressure and cardiac frequency, before and after 8 weeks of the VLCKD (Table 3). 

**Figure 1:**
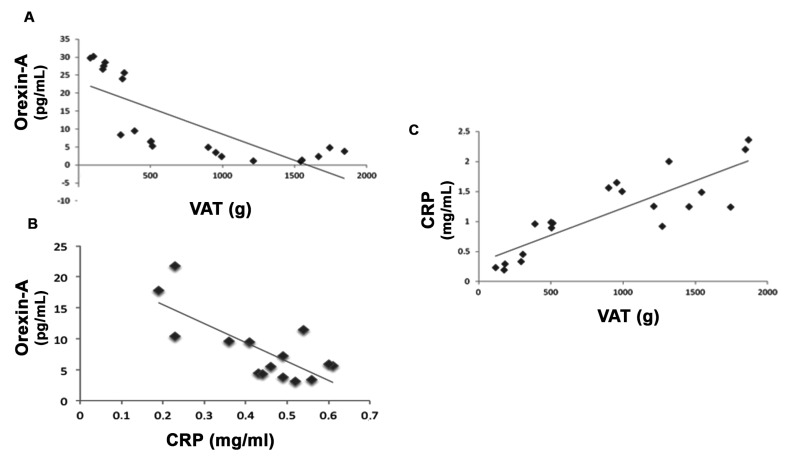
VAT strongly correlates with orexin-A and C-reactive protein (CRP) serum levels in very low calorie ketogenic diet (VLCKD) subjects before and after diet. Δ-variation in VLCKD subjects before and after diet showed a negative correlation between visceral adipose tissue (VAT) and orexin-A serum levels (**A**) and between orexin-A and CRP serum levels (**B**) in VLCKD subjects. Furthermore, there was a positive correlation between VAT and CRP serum levels in VLCKD subjects both before and after diet (**C**).

In this study, we also investigated the inflammatory state of subjects before and after dietary intervention. Table 1 shows that the orexin-A and CPR levels seem to be strongly modulated before and after VLCKD treatment. Interestingly, the orexin-A levels in subjects after diet were statistically increased and CRP levels were also strongly reduced after the VLCKD compared with before diet. Moreover, there appear to be statistical correlations between VAT and orexin-A, VAT and CRP levels, and orexin-A and CRP levels in VLCKD subjects, both before and after VLCKD therapy (Figure 1).

### 3.2. MTT Test

**Figure 2:**
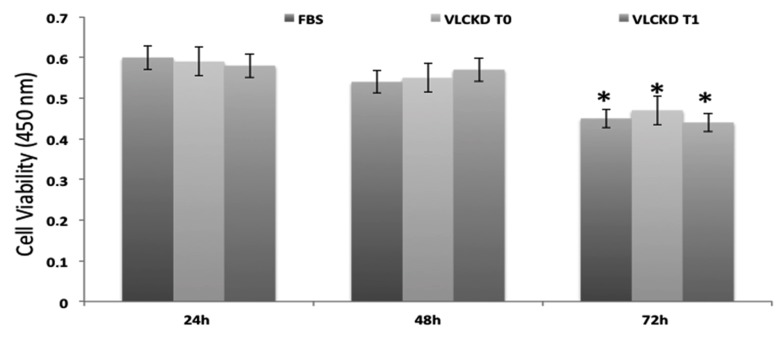
Cytotoxic effects of obese subjects’ sera before and after 8 weeks of the VLCKD. The cell viability of Hep-G2 cells cultured and treated for 24, 48, and 72 h with VLCKD obese subjects’ sera before and after 8 weeks of diet was not reduced compared to Hep-G2 cells treated with only fetal bovine serum (FBS) used as controls. *: statistically significant difference.

To investigate the effects on cell viability using the sera from VLCKD subjects before and after diet, we treated Hep-G2 cells for 24, 48, and 72 h with these sera and evaluated cell proliferation using the MTT assay kit. As a control, we used cells incubated with 10% FBS. Our results show that cell viability was not affected by the treatment with sera before and after the VLCKD after 24, 48, and 72 h of incubation, but we found a cell viability decrease after 72 h of incubation compared with 24 and 48 h of incubation, both with FBS and VLCKD sera before and after diet. These cytotoxic effects were not caused by the treatment but depend on time of incubation (Figure 2). 

### 3.3. ROS Production

**Figure 3:**
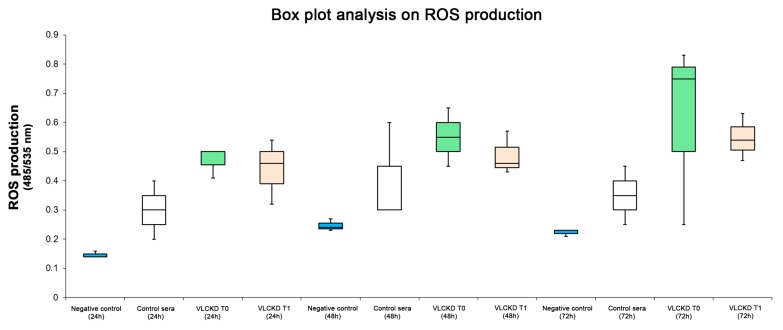
Reactive oxygen species (ROS) production was strongly modulated before and after the VLCKD. Hep-G2 cells cultured and treated for 24, 48, and 72 h with only FBS and with VLCKD obese subjects’ sera before and after 8 weeks of diet showed a statistically significant increase of ROS production compared with Hep-G2 cells treated with N-acetylcysteine (NAC) for 24, 48, and 72 h.

**Figure 4:**
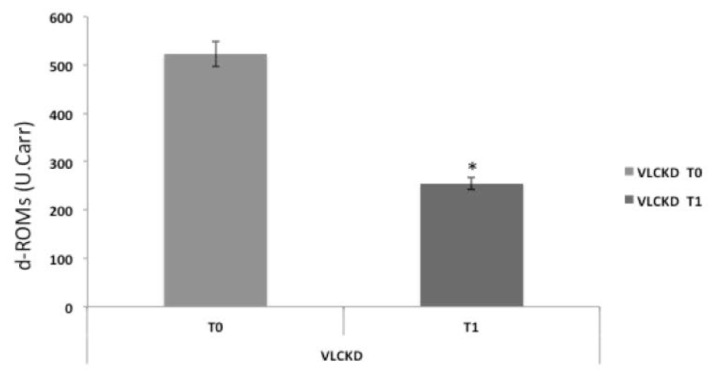
Reactive oxygen metabolite (d-ROM) plasma levels were strongly modified in VLCKD subjects. Reactive oxygen metabolites in the plasma of VLCKD obese subjects before diet were higher than those of VLCKD obese subjects after diet therapy. *: statistically significant difference.

The level of ROS was analyzed in cells treated with 5% (*v/v*) pooled sera from VLCKD subjects, collected prior and after dietary ketosis; moreover, it was tested in cells treated with 5% pooled sera from healthy subjects (matched with controls for age and sex) and compared to additional NAC treatment (an ROS inhibitor) used as a negative control. As shown in the box plot analysis (Figure 3), there was ROS production in cells treated with pooled sera from healthy subjects and pooled sera from VLCKD obese subjects before and after diet compared to cells treated with only NAC. Furthermore, there was a statistical increase of ROS production in cells treated with VLCKD obese subjects’ sera before and after diet compared with cells treated with pooled sera from healthy subjects (Figure 4). Nonetheless, in VLCKD obese subjects, there was a difference in ROS production before and after diet therapy. In particular, there was a reduction of ROS levels in Hep-G2 cells treated with VLCKD subjects’ sera after diet, with no statistically significant reduction. 

### 3.4. Serum Reactive Oxygen Metabolites Test 

We analyzed reactive oxygen metabolites in plasma of VLCKD obese subjects before and after diet using the d-ROM test. The results of the d-ROM test showed different values: in the subjects before the VLCKD, the value was statistically higher than in subjects after the VLCKD (532 U vs. 255 U, *p < 0.05*). This finding suggests a decrease of oxidative stress in VLCKD subjects after diet. 

## 4. Discussion 

The main findings of the present study may be summarized in terms of a decrease in weight, BMI, fat mass, and visceral adipose tissue and an increase in orexin-A serum levels after 8 weeks of diet intervention.

Moreover, in vitro studies have shown that this dietary intervention does not have cytotoxic effects on Hep-G2 cell lines. Furthermore, our results demonstrate that the VLCKD has numerous beneficial effects both on anthropometric and biochemical parameters. Furthermore, the weight loss and BMI reduction that we observed in our subjects were associated with a strong reduction of visceral adipose tissue. As supported by Moreno et al., the reduction of visceral fat leads to a lowering of cardiovascular disease, diabetes, and even several kinds of cancer risk [24]. Visceral fat mass creates chronic low inflammation through an imbalance of the production of adipokines by adipose tissue. Aaron et al. hypothesized that chronic adipose-tissue-driven inflammation influences lymphatic tissue function, in part, by changes in resident immune cell populations [25,26]. 

For these reasons, the reduction of VAT leads to a decrease of chronic inflammation, blocking proinflammatory cytokines and enhancing anti-inflammatory production, as supported by a decrease of CRP in the serum of VLCKD subjects after diet intervention. Indeed, as supported by Abraham et al., in visceral fat accumulation, typical of an obese condition, CRP is released from the liver in response to the inflammatory state, which represents a risk factor for cardiovascular events and is related to an increased risk of diabetes type 2 [27]. Moreover, it is well known that reducing visceral fat mass induces increases in lean mass and skeletal bone preservation. These noteworthy findings tend to reinforce the beneficial effect of a VLCK diet in obesity treatment, although, as reported by Moro et al., it could be sufficient to practice caloric restriction to improve some health-related biomarkers, decrease fat mass, and maintain muscle mass in resistance-trained subjects [28]. Furthermore, as reported by Kosinski et al., very low carbohydrate diets can be different in macronutrient composition and high-fat versus high-protein content [29]. Moreover, Fung et al. reported that the ketogenic diet may be preferred with a high amount of vegetables in order to decrease cardiovascular-related mortality [30]. In addition to amelioration of anthropometric parameters, our results also showed an improvement in glycemic and lipid profiles, reducing insulinemia, glycated hemoglobin, triglycerides, and LDL cholesterol. 

In general, the VLCK diet has numerous beneficial effects on various organs and tissues. This diet requires a significant restriction of both calories and carbohydrates, which induces a reduction in glycogen stores in both the liver and muscles, induces metabolic ketosis, and reduces glucose production and liver secretion, lowering blood glucose levels as its main effects. Indeed, insulin resistance in the liver and muscles dissipates as the fat stores in these organs are reduced. The VLCKD also increases pancreatic insulin sensitivity through mechanisms related to and independent of ketosis. Finally, the ketogenic state improves satiety, which leads to greater dietary compliance and tends to improves hyperlipidemia [31]. In agreement with our results, Bueno et al. reported that very low carbohydrate diets induce good weight loss, an improvement in lipid profiles, and a reduction in diastolic blood pressure [32]. On the contrary, some studies on long-term VLCKD reported that this diet intervention may have negative effects on lipid profiles, increasing LDL cholesterol and reducing HDL cholesterol [33]. In addition, the VLCKD also has beneficial effects on brain function. Interestingly, the mechanisms of action of the VLCKD seem to go far beyond the regulation of neurotransmitters. As previously described [34], in vivo studies indicate that the VLCKD is associated with vascular brain changes, increasing vascular density at the blood–brain barrier without changes in blood flow. It has been hypothesized that increasing capillary density due to the high plasmatic ketone body rate would enhance up to 40-fold the flux of ketonic bodies available for cerebral energetic metabolism [34], suggesting that a similar mechanism may be involved in improving energetic supply to the brain during starvation [34].

The present study also evaluated the ORX1 plasmatic levels, which were strongly increased in VLCKD obese subjects after 8 weeks of dietary treatment. This hypothalamic neuropeptide may be considered as “multitasking” considering its involvement in weight loss, energy homeostasis, cognition, mood, and sleep regulation [35]. According to our results, plasmatic orexin-A levels are strongly reduced in obese subjects and seem to be inversely correlated to proinflammatory mediators and BMI [13]. Shiuchi et al. observed that the regulation mediated by the orexinergic system on muscle glucose metabolism is due to the activation of β2-adrenergic signaling and, consequently, peripheral energy expenditure [36]. 

We also have to highlight the relevance of obesity as a proinflammatory condition, as shown by the d-ROM serum level decrease after 8 weeks of VLCKD in obese subjects. Many reports have supported that adipose tissue contributes to systemic lipid peroxides directly linked to adiposity both in humans and rodents [37,38,39,40]. 

On the other hand, considering that visceral adipose tissue quantity seems to be more strongly associated with oxidative stress levels than abdominal subcutaneous fat, we analyzed the effects of the VLCKD on hepatocyte cell cultures to evaluate the possible cytotoxic effects and ROS production. As reported in Figure 2, the cell viability of Hep-G2 cultures treated with sera of obese patients before and after 8 weeks of diet was not reduced compared to Hep-G2 cells treated with only FBS as a control, and ROS production was statistically significantly reduced in cell cultures treated with VLCKD sera after 8 weeks, suggesting the lack of cytotoxic effects due to VLCKD intervention in these patients. This result may be interpreted not only with ameliorating anthropometric and biochemical parameters but also with the VLCKD inducing the expression of many genes involved in cell repair reducing the expression of genes involved in the mechanisms of oxidative stress and inflammation [41,42,43]. On the other hand, Ziegler et al., reported that correct nutrition and regular physical activity are able to activate numerous metabolic pathways such as SIRT1, which deacetylates nuclear and cytoplasmic proteins controlling the apoptotic processes, downregulating the production of flogistic mediators [44]. 

In light of what has been stated above, Greco et al. reported that the ketogenic diet activates the Nrf2/ARE system in both acute and chronic settings, protecting cells from oxidative stress and apoptotic signals induced by the upregulating expression of NQO1, SOD1, and SOD2, which are antioxidant proteins [45]. 

In the obese condition, exposure to ROS also induces a reduction in the antioxidant capacity in the liver, which increases the possibility of cell damage by oxidative stress. ROS in a physiological state have many beneficial functions, such as gene expression, cell growth, defense against infections, and the control of vascular endothelial cells. However, when there is excessive ROS production, such as in obesity, cells are exposed to oxidative stress and are damaged. In this scenario, VLCKD intervention may represent a strategy against an excess of ROS. There are many reports that the VLCKD tends to reduce ROS production, increasing SOD1 and SOD2, and induces the reduction of proinflammatory cytokine production, increasing anti-inflammatory mediators [46]. 

## 5. Conclusions

We can conclude that the VLCKD exerts a positive effect on VAT reduction, ameliorating adiposity and blood biochemical parameters. In the short term, this dietary intervention reduced inflammation and ROS production. Finally, to the best of our knowledge, this is the first study to report the effects of the VLCKD on the orexinergic system, supporting the usefulness of such a therapeutic intervention in promoting the reduction of the individual burden of this disease.

## Figures and Tables

**Table 1:** Anthropometric and biochemical parameters of very low calorie ketogenic diet (VLCKD) subjects before and after weight loss.

Parameters	VLCKD Obese Subjects		Statistical Analysis
	**T0**	**T1**	***p*-Value**
Age	48 ± 8.2		ns
Height (m)	1.67 ± 0.11		ns
Weight (kg)	91.33 ± 17.11	78.73 ± 13.36	<0.001
BMI (kg/m^2^)	32.19 ± 4.78	27.76 ± 3.62	<0.001
TOTAL CHOLESTEROL (mg/dL)	220.13 ± 50.77	173.91 ± 32.93	<0.05
HDL (mg/dL)	55.13 ± 11.14	47.76 ± 9.14	ns
LDL (mg/dL)	141.83 ± 36.48	107.57 ± 27.72	<0.05
TRIGLYCERIDES (mg/dL)	135.54 ± 125.27	83.25 ± 26.14	<0.05
TOTAL BILIRUBIN (mg/dL)	0.61 ± 0.22	0.68 ± 0.27	ns
DIRECT BILIRUBIN (mg/dL)	0.13 ± 0.18	0.16 ± 0.09	ns
INDIRECT BILIRUBIN (mg/dL)	0.48 ± 0.33	0.52 ± 0.21	ns
HEMOGLOBIN (g/dL)	14.13 ± 1.33	13.83 ± 0.94	ns
GLYCATED HEMOGLOBIN (Hba1c) (%)	5.65 ± 0.39	5.38 ± 0.33	ns
INSULINEMIA (uUl/mL)	10.53 ± 7.18	5.37 ± 3.79	<0.05
URIC ACID (mg/dL)	4.86 ± 1.01	5.27 ± 1.13	ns
TOTAL PROTEINS (g/dL)	7.30 ± 0.4	7.13 ± 0.4	ns
SERUM GLUTAMIC OXALOACETIC TRANSAMINASE (U/L)	21.27 ± 5.98	23.31 ± 11.47	<0.05
SERUM GLUTAMIC PYRUVIC TRANSAMINASE (U/L)	26.51 ± 14.89	26.06 ± 16.27	<0.05
GAMMA GLUTAMYL TRANSFERASE (U/L)	31.19 ± 19.88	15.31 ± 5.41	<0.05
AZOTEMIA (mg/dL)	35.35 ± 8.43	34.68 ± 9.16	ns
CALCEMIA (mg/dL)	9.57 ± 0.33	9.72 ± 0.35	ns
SODIUM (mmol/L)	139.19 ± 2.48	139.18 ± 2	ns
C-REACTIVE PROTEIN (mg/mL)	0.89 ± 0.1	0.48 ± 0.07	<0.05
Orexin-A (pg/mL)	9.91 ± 0.27	16.24 ± 1.33	<0.001

T0: values before diet; T1: values after diet; ns: not significant (over 0.05).

**Table 2:** Body composition of VLCKD subjects before and after weight loss.

Parameters	VLCKD Obese Subjects		Statistical Analysis
	**T0**	**T1**	***p*-Value**
Visceral Adipose Tissue (VAT) (g)	1541.55 ± 141.63	927.79 ± 104.92	<0.001
Fat Mass (FM) (g)	39,208.77± 1432.55	27377.0 ± 1217.48	<0.001
FFM (g)	48,789.57± 1712.36	48093.68 ± 1670.65	ns
BMD	1225.57 ± 21.23	1229.31 ± 21.46	ns

FFM: fat-free mass; BMD: bone mineral density; ns: not significant (over 0.05).

**Table 3:** Cardiac parameters of VLCKD subjects before and after weight loss.

Parameters	VLCKD Obese Subjects		Statistical Analysis
	**T0**	**T1**	***p*-Value**
SYSTOLIC ARTERIAL PRESSURE	130.6	117. 5	<0.001
DIASTOLIC ARTERIAL PRESSURE	82. 8	75.8	<0.001
CARDIAC FREQUENCY PRE 6MWT	77.7	74.5	<0.001
CARDIAC FREQUENCY POST 6MWT	142.6	138.4	<0.001

6MWT: 6 Minutes Walking Test.

## References

[1] Bray G.A., Kim K.K., Wilding J.P.H. (2017). Obesity: A chronic relapsing progressive disease process. A position statement of the World Obesity Federation. Obes. Rev..

[2] Joint WHO/FAO Expert Consultation (2003). World Health Organization: Diet., Nutrition and the Prevention of Chronic Diseases.

[3] Polito R., Nigro E., Messina A., Monaco M.L., Monda V., Scudiero O., Cibelli G., Valenzano A., Picciocchi E., Zammit C. (2018). Adiponectin and Orexin-A as a Potential Immunity Link Between Adipose Tissue and Central Nervous System. Front. Physiol..

[4] Ibrahim M.M. (2010). Subcutaneous and visceral adipose tissue: Structural and functional differences. Obes. Rev..

[5] Alexopoulos N., Katritsis D., Raggi P. (2014). Visceral adipose tissue as a source of inflammation and promoter of atherosclerosis. Atherosclerosis.

[6] Bi X., Seabolt L., Shibao C., Buchowski M., Kang H., Keil C.D., Tyree R., Silver H.J. (2015). Dxa-measured visceral adipose tissue predicts impaired glucose tolerance and metabolic syndrome in obese Caucasian and African-American women. Eur. J. Clin. Nutr..

[7] Hauner H. (2005). Secretory factors from human adipose tissue and their functional role. Proc. Nutr. Soc..

[8] Halberg N., Wernstedt-Asterholm I., Scherer P.E. (2008). The adipocyte as an endocrine cell. Endocrinol. Metab. Clin. N. Am..

[9] Messina A., Monda V., Sessa F., Valenzano A., Salerno M., Bitetti I., Precenzano F., Marotta R., Lavano F., Lavano S.M. (2018). Sympathetic, Metabolic Adaptations, and Oxidative Stress in Autism Spectrum Disorders: How Far from Physiology?. Front. Physiol..

[10] Roccella M., Marotta R., Operto F.F., Smirni D., Precenzano F., Bitetti I., Messina G., Sessa F., Di Mizio G., Loreto C. (2019). NREM Sleep Instability in Pediatric Migraine without Aura. Front. Neurol..

[11] Tsujino N., Sakurai T. (2009). Orexin/hypocretin: A neuropeptide at the interface of sleep, energy homeostasis, and reward system. Pharmacol. Rev..

[12] Messina G., Dalia C., Tafuri D., Monda V., Palmieri F., Dato A., Russo A., De Blasio S., Messina A., De Luca V. (2014). Orexin-A controls sympathetic activity and eating behavior. Front. Psychol..

[13] Alam M.N., Kumar S., Bashir T., Suntsova N., Methippara M.M., Szymusiak R. (2005). GABA-mediated control of hypocretin- but not melanin-concentrating hormone-immunoreactive neurones during sleep in rats. J. Physiol..

[14] Adam J.A., Menheere P.P.C.A., van Dielen F.M.H., Soeters P.B., Buurman W.A., Greve J.W.M. (2002). Decreased plasma orexin-A levels in obese individuals. Int. J. Obes..

[15] Digby J.E., Chen J., Tang J.Y., Lehnert H., Matthews R.N., Randeva H.S. (2006). Orexin receptor expression in human adipose tissue: Effects of orexin-A and orexin-B. J. Endocrinol..

[16] Blais A., Drouin G., Chaumontet C., Voisin T., Couvelard A., Even P.C. (2017). Impact of Orexin-A Treatment on Food Intake, Energy Metabolism and Body Weight in Mice. PLoS ONE.

[17] Kok S.W., Overeem S., Visscher T.L.S., Lammers G.J., Seidell J.C., Pijl H. (2003). Hypocretin deficiency in narcoleptic humans is associated with abdominal obesity. Obes. Res..

[18] Salminen A., Kaaeniranta A., Kauppinen A. (2013). Crosstalk between Oxidative Stress and SIRT1: Impact on the Aging Process. Int. J. Mol. Sci..

[19] Boison D. (2017). New insights into the mechanisms of the ketogenic diet. Curr. Opin. Neurol..

[20] Adam-Perrot A., Clifton P., Brouns F. (2006). Low carbohydrate diets: Nutritional and physiological aspects. Obes. Rev..

[21] Micklesfield L.K., Goedecke J.H., Punyanitya M., Wilson K.E., Kelly T.L. (2012). Dual-energy X-ray performs as well as clinical computed tomography for the measurement of visceral fat. Obesity.

[22] (2002). SCOOP-VLCD Task 7.3 Reports on Tasks for Scientific Cooperation.

[23] Cornelli U., Terranova R., Luca S., Cornelli M., Alberti A. (2001). Bioavailability and antioxidant activity of some food supplements in men and women using the D-Roms test as a marker of oxidative stress. J. Nutr..

[24] Moreno B., Crujeiras A.B., Bellido D., Sajoux I., Casanueva F.F. (2016). Obesity treatment by very low-calorie-ketogenic diet at two years: Reduction in visceral fat and on the burden of disease. Endocrine.

[25] Farb M.G., Gokce N. (2015). Visceral adiposopathy: A vascular perspective. Horm. Mol. Biol. Clin. Investig..

[26] Magnuson A.M., Fouts J.K., Regan D.P., Booth A.D., Dow S.W., Foster M.T. (2018). Adipose Tissue Extrinsic Factor: Obesity-Induced Inflammation and the Role of the Visceral Lymph Node. Physiol. Behav..

[27] Abraham P.A., Attipoe S., Kazman J.B., Zeno S.A., Poth M., Deuster P.A. (2017). Role of plasma adiponectin/C-reactive protein ratio in obesity and type 2 diabetes among African Americans. Afr. Health Sci..

[28] Moro T., Tinsley G., Bianco A., Marcolin G., Pacelli Q.F., Battaglia G. (2016). Effects of eight weeks of time-restricted feeding (16/8) on basal metabolism, maximal strength, body composition, inflammation, and cardiovascular risk factors in resistance-trained males. J. Transl. Med..

[29] Kosinski C., Jornayvaz F.R. (2017). Effects of Ketogenic Diets on Cardiovascular Risk Factors: Evidence from Animal and Human Studies. Nutrients.

[30] Fung T.T., van Dam R.M., Hankinson S.E., Stampfer M., Willett W.C., Hu F.B. (2010). Low-carbohydrate diets and all-cause and cause-specific mortality: Two cohort studies. Ann. Intern. Med..

[31] Scott S.N., Anderson L., Morton J.P., Wagenmakers A.J.M., Riddell M.C. (2019). Carbohydrate Restriction in Type 1 Diabetes: A Realistic Therapy for Improved Glycaemic Control and Athletic Performance?. Nutrients.

[32] Bueno N.B., de Melo I.S., de Oliveira S.L., da Rocha A. (2013). Very-low-carbohydrate ketogenic diet v.low-fat diet for long-term weight loss: A meta-analysis of randomised controlled trials. Br. J. Nutr..

[33] Kwiterovich P.O., Vining E.P., Pyzik P., Skolasky R., Freeman J.M. (2003). Effect of a High-Fat Ketogenic Diet on Plasma Levels of Lipids, Lipoproteins, and Apolipoproteins in Children. JAMA.

[34] Puchowicz M.A., Zechel J., Valerio J., Emancipator D., Xu K., Pundik S., LaManna J.C., Lust D. (2008). Neuroprotection in Diet Induced Ketotic Rat Brain Following Focal Ischemia. J. Cereb. Blood Flow Metab..

[35] Shiuchi T., Haque M.S., Okamoto S., Inoue T., Kageyama H., Lee S. (2009). Hypothalamic orexin stimulates feeding-associated glucose utilization in skeletal muscle via sympathetic nervous system. Cell Metab..

[36] Chieffi S., Carotenuto M., Monda V., Valenzano A., Villano I., Precenzano F. (2017). Orexin System: The Key for a Healthy Life. Front. Physiol..

[37] Coborn J.E., Deporter D.P., Mavanji V., Sinton C.M., Kotz C.M., Billington C.J. (2017). Role of orexin-a in the ventrolateral preoptic area on components of total energy expenditure. Int. J. Obes..

[38] Williams R.H., Alexopoulos H., Jensen L.T., Fugger L., Burdakov D. (2008). Adaptive sugar sensors in hypothalamic feeding circuits. Proc. Natl. Acad. Sci. USA.

[39] Furukawa S., Fujita T., Shimabujuro M. (2004). Increased oxidative stress in obesity and its impact on metabolic syndrome. J. Clin. Investig..

[40] Gletsu Miller N., Hansen J.M., Jones D.P., Go Y.M., Torres W.E., Ziegler T.R., Lin E. (2009). Loss of Total and Visceral Adipose Tissue Mass Predicts Decreases in Oxidative Stress After Weight Loss Surgery. Obesity.

[41] Cancello R., Henegar C., Viguerie N. (2005). Reduction of macrophage infiltration and chemoattractant gene expression changes in white adipose tissue of morbidly obese subjects after surgery-induced weight loss. Diabetes.

[42] Clement K., Viguerie N., Poitou C. (2004). Weight loss regulates inflammation-related genes in white adipose tissue of obese subjects. FASEB J..

[43] Ziegler D.R., Ribeiro L.C., Hagenn M. (2003). Ketogenic diet increases glutathione peroxidase activity in rat hippocampus. Neurochem. Res..

[44] Venugopal R., Jaiswal A.K. (1996). Nrf1 and Nrf2 positively and c-Fos and Fra1 negatively regulate the human antioxidant response element-mediated expression of NAD(P)H:quinone oxidoreductase1 gene. Proc. Natl. Acad. Sci. USA.

[45] Greco T., Glenn T., Hoyda D., Prins L.M. (2016). Ketogenic diet decreases oxidative stress and improves mitochondrial respiratory complex activity. J. Cereb. Blood Flow Metab..

[46] Jornayvaz F.R., Jurczak M.J., Lee H.-Y., Birkenfeld A.L., Frederick D.W., Zhang D., Zhang X.M., Samuel V.T., Shulman G.I. (2010). A high-fat, ketogenic diet causes hepatic insulin resistance in mice, despite increasing energy expenditure and preventing weight gain. Am. J. Physiol. Endocrinol. Metab..

